# 927. Cidofovir in the Treatment of BK Virus–Associated Hemorrhagic Cystitis Following Hematopoietic Stem Cell Transplantation; A Medication Use and Safety Analysis

**DOI:** 10.1093/ofid/ofad500.972

**Published:** 2023-11-27

**Authors:** Ryan Nazareno, Dhaval Patel, Lea M Monday

**Affiliations:** Wayne State University School of Medicine, Flat Rock, Michigan; Karmanos Cancer Institute, Detroit, Michigan; Wayne state University School of Medicine, Detroit, Michigan

## Abstract

**Background:**

BK virus hemorrhagic cystitis (BKV-HC) is a complication after allogeneic hematopoietic stem cell transplant (AlloHCT) for which optimal management is uncertain. Intravenous (IV) and intravesicular (IVES) cidofovir have been used with varying degrees of success in small case series of six to 33 patients. While some series have investigated side effects, none have examined medication errors (Fig 1).

**Figure:**
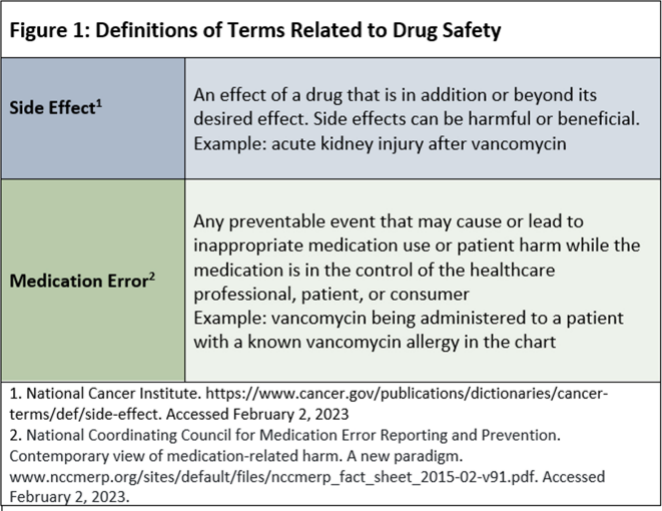

**Methods:**

A retrospective single-center case series of AlloHCT patients with BKV-HC given IV or IVES cidofovir (CDV) from 2018 to 2022 at an urban cancer center in Detroit, MI. Our primary objective was to determine the incidence of CDV related medication errors (ME) and perform a root cause analysis (RCA) to determine factors contributing to such events. Secondary objectives were to describe the effectiveness of CDV for BKV-HC following AlloHCT.

**Results:**

Six AlloHCT patients were treated with CDV. Patient characteristics are shown in Table 1. Median age was 48 years (range 32-63), with most being high risk for cytomegalovirus (R+) and experiencing acute graft versus host disease. CDV and BKV-HC characteristics are shown in Table 2. BKV-HC occurred a median of 53 days post Allo-HCT (range 27-179). Four patients received IV CDV only and two received it both IV and IVES. Median number of doses was 2 (range 1-10). Median BKV-HC severity grade was 2.5, and three of six patients had BK viremia (Table 2). Five of six patients had microscopic resolution of hematuria (median time to resolution 30 days, range 1-116). However, 4 of 6 had died and 1 of 6 had recurrence of BKV-HC within 90 days. The most common CDV side effects were bladder pain/spasms (n=5) and acute kidney injury (n=4). There were 2 MEs; one near miss where CDV was incorrectly ordered IV but changed to IVES by a pharmacist, and one major safety event where an IVES dose was administered IV. RCA analysis revealed multiple contributing factors including similarity in appearance of doses and an overly simplified pump library without an option for IVES administration (Fig 2).

**Figure:**
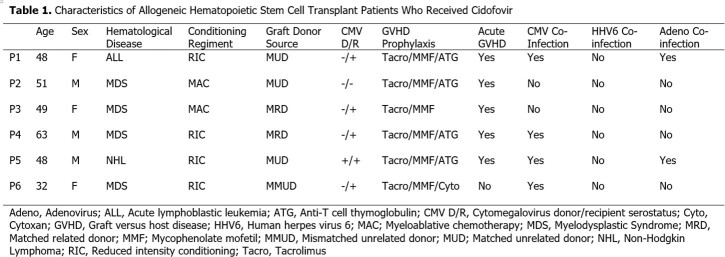

**Figure:**
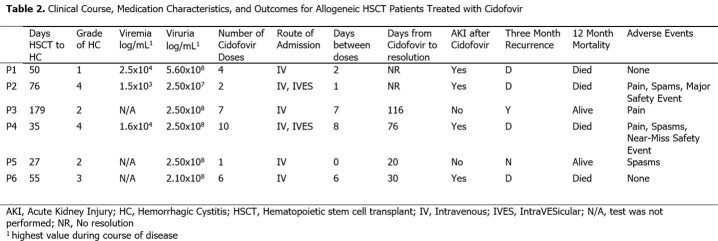

**Figure:**
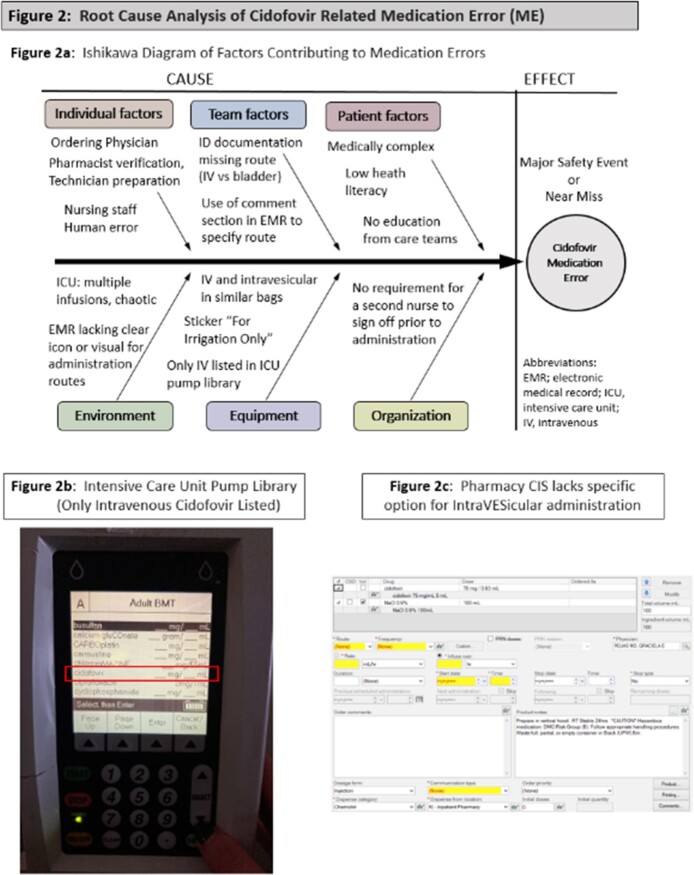

**Conclusion:**

In this first case series to describe medication errors in patients treated with CDV for BKV-HC, one in three patients experienced an ME. Clinicians caring for AlloHCT patients should have a high predisposition for error when cidofovir is being prescribed.

**Disclosures:**

**All Authors**: No reported disclosures

